# Use of Pessaries, Etc.

**Published:** 1880-10-20

**Authors:** I. T. Suggs


### USE OF PESSARIES, ETC.

BY I. T. SUGGS, M.D.

In reply to Dr. Gibson's request, relative to pessaries, I send you the following :

In the first place, what causes prolapsus uteri ? When we consider the size, position, supports of the healthy uterus, we see at a glance that such a condition as prolapsus is almost, if not altogether, impossible unless there is inflammation, abrasion or ulceration, and consequently an increase of size and weight. Thus, prolapsus is not the disease, but a symptom or perhaps the result of disease. Such being the case, the hard pessary coming in contact with the already diseased os uteri, certainly does more harm than good; such, I am satisfied, is the result of my experience. Since the introduction of the metroscopic treatment which I adopted in the year 1853, I have in my treatment of uterine diseases used the ring pessary with much benefit. It does not in any way irritate the diseased os; the sponge pessary has in some cases proven serviceable. I have for the last 27 years made the treatment of chronic female diseases a specialty, so much so as could be in a country practice, and am satisfied that chronic uterine diseases are as curable a class of diseases as I have ever attended. Cure the disease, and the prolapsus disappears. An outline of my treatment has been as follows such constitutional treatment, as the symptoms required; especially regulate the bowels and keep them so; use tonics as may be indicated. Local treatment : Caustics of different kinds, nitrate of silver, caustic potassa, iodine, acid nitrate of mercury, carbolic acid, iodine and carbolic acid, etc., with various washes, as carbolic acid diluted, alum, acetate of lead, cleansing washes, especially at night if there is much vaginal discharge, oftener if required. Avoid all stooping or straining work; take plenty of moderate exercise. In indolent ulcer, where there is albuminous discharge, use a small cup-shaped sponge, applied to the os, filled with brown sugar and salt; let it remain 8 or 10 hours; attach a small cord by which she can remove it. This generally increases the discharge. Do not discharge the patient under two months after the disease is apparently well; still see her occasionally for four or five weeks, so as to be sure she is permanently cured.

